# A Guide to Literature Informed Decisions in the Design of Real Time fMRI Neurofeedback Studies: A Systematic Review

**DOI:** 10.3389/fnhum.2020.00060

**Published:** 2020-02-25

**Authors:** Samantha J. Fede, Sarah F. Dean, Thushini Manuweera, Reza Momenan

**Affiliations:** Clinical NeuroImaging Research Core, National Institute on Alcohol Abuse and Alcoholism, National Institutes of Health, Bethesda, MD, United States

**Keywords:** neurofeedback, fMRI, methods, rt-fMRI, intervention

## Abstract

**Background:** Although biofeedback using electrophysiology has been explored extensively, the approach of using neurofeedback corresponding to hemodynamic response is a relatively young field. Real time functional magnetic resonance imaging-based neurofeedback (rt-fMRI-NF) uses sensory feedback to operantly reinforce patterns of neural response. It can be used, for example, to alter visual perception, increase brain connectivity, and reduce depression symptoms. Within recent years, interest in rt-fMRI-NF in both research and clinical contexts has expanded considerably. As such, building a consensus regarding best practices is of great value.

**Objective:** This systematic review is designed to describe and evaluate the variations in methodology used in previous rt-fMRI-NF studies to provide recommendations for rt-fMRI-NF study designs that are mostly likely to elicit reproducible and consistent effects of neurofeedback.

**Methods:** We conducted a database search for fMRI neurofeedback papers published prior to September 26th, 2019. Of 558 studies identified, 146 met criteria for inclusion. The following information was collected from each study: sample size and type, task used, neurofeedback calculation, regulation procedure, feedback, whether feedback was explicitly related to changing brain activity, feedback timing, control group for active neurofeedback, how many runs and sessions of neurofeedback, if a follow-up was conducted, and the results of neurofeedback training.

**Results:** rt-fMRI-NF is typically upregulation practice based on hemodynamic response from a specific region of the brain presented using a continually updating thermometer display. Most rt-fMRI-NF studies are conducted in healthy samples and half evaluate its effect on immediate changes in behavior or affect. The most popular control group method is to provide sham signal from another region; however, many studies do not compare use a comparison group.

**Conclusions:** We make several suggestions for designs of future rt-fMRI-NF studies. Researchers should use feedback calculation methods that consider neural response across regions (i.e., SVM or connectivity), which should be conveyed as intermittent, auditory feedback. Participants should be given explicit instructions and should be assessed on individual differences. Future rt-fMRI-NF studies should use clinical samples; effectiveness of rt-fMRI-NF should be evaluated on clinical/behavioral outcomes at follow-up time points in comparison to both a sham and no feedback control group.

## Introduction

Biofeedback training (based largely on EMG and EKG readings) has been established as an efficacious intervention for a variety of medical conditions, including hypertension, headaches, chronic pain, and female urinary incontinence (Yucha and Montgomery, 2008). Neurofeedback is a form of biofeedback in which information about neural processes is given to an individual. EEG-based neurofeedback is also well-established as an effective intervention for ADHD, epilepsy, and anxiety.

As we understand more about how patterns of neural activity underly behavior and disorder, the pie in the sky goal for neurofeedback training is to be able to non-invasively change these patterns and address a variety of largely intractable neurological and mental health conditions. EEG-based neurofeedback is well-established, but its limitations in anatomical specificity have led researchers to modalities like real-time functional magnetic resonance imaging (rt-fMRI) to address more complex behavioral disorders.

## How Does Neurofeedback Work?

There are two mechanisms by which biofeedback, including neurofeedback, is thought to work (Frank et al., 2010). The first is operant conditioning, which is a model of learning where we modify our behaviors based on observing the consequences. When patients see their physiological or neural response, they are able to identify on an implicit level what their response at different levels feels like. When guided to practice regulating that response to a healthier level, patients operantly learn how to volitionally change that response. The second mechanism is complimentary; by identifying cognitive behavioral strategies that result in visible improvements, or by identifying those behaviors or thought processes that result in negative patterns, patients can learn to engage in those strategies, or avoid those elements, in the absence of feedback.

**Figure 1:**
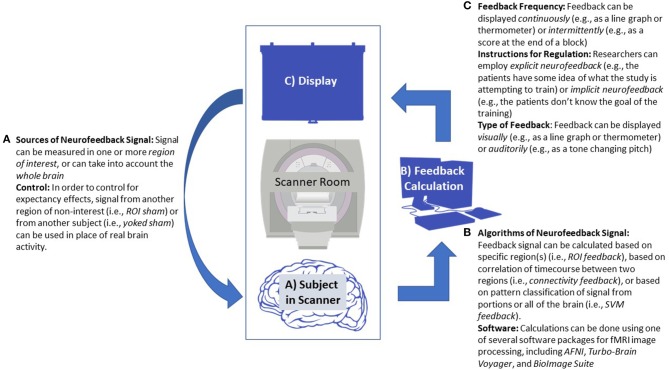
Diagram of a generic neurofeedback experiment with study design elements highlighted. **(A)** Functional MRI data is collected from a patient, focusing either on a single region of interest (ROI), multiple ROIs, or the whole brain. **(B)** Data from the subject is processed in real-time, usually employing standard neuroimaging software packages. Feedback is calculated, sometimes based on ROI signal change, correlation between several ROIs, or using a classification algorithm. This may require an offline fMRI scan prior to neurofeedback training to establish baseline activity or to train the classifier. **(C)** The feedback value is transformed and given to the patient in the scanner, often via a continuous visual graph displayed on a screen. **(A)** This allows the participant to react and change their neural response, starting the feedback cycle over.

Rt-fMRI neurofeedback (rt-fMRI-NT) works by providing a signal representing hemodynamic response in a given portion of the brain. See Figure 1 for a diagram of a typical neurofeedback setup. Considering the physiology underlying that response, there is necessarily a delay between the desired neural firing experimenters hope to reinforce, as opposed to the several hundred millisecond level of EEG neurofeedback. However, an advantage to rt-fMRI based neurofeedback is the specificity through which it can reinforce engagement or regulation of specific parts of the brain (Ramot et al., 2017). Moreover, insights from functional near-infrared spectroscopy neurofeedback studies suggest that part of the mechanism by which neurofeedback based on hemodynamics works is by focusing neural response during the desired behavior to the target region of interest (ROI; Kober et al., 2014; Barth et al., 2016).

The field of rt-fMRI-NF has boomed in recent years, likely overcoming some of the logistic difficulties associated with conducting rt-fMRI neurofeedback studies (e.g., computer processing power, costs) and evidence of its safety (Hawkinson et al., 2012). However, there is still much skepticism regarding the practicality of rt-fMRI neurofeedback as a clinical intervention, given the relative costs and inconveniences of MRI for patients and the lack of clear treatment efficacy (Thibault et al., 2018), particularly compared to cheaper options like medication and psychotherapy. As we begin new rt-fMRI neurofeedback experiments, we owe it to both patients and the field to make our best effort to ensure our studies are optimally designed and powered to detect and characterize the effects of rt-fMRI neurofeedback on behavior and symptomology. However, there is little consensus in the field as to what these best practices are. This systematic review aims to provide some grounds for this standardization. We approach this by first summarizing the work on neurofeedback in terms of psychological domains and what clinical disorders have been investigated. Then, we review various design elements of neurofeedback studies and different ways they have been implemented in the prior literature. Each section is then concluded by a recommendation based on the critical summary of the evidence, providing guidelines based on the relative efficacy of each approach. Finally, we discuss other considerations for implementing neurofeedback, including individual differences, and software options.

## Systematic Review Methods

**Figure 2:**
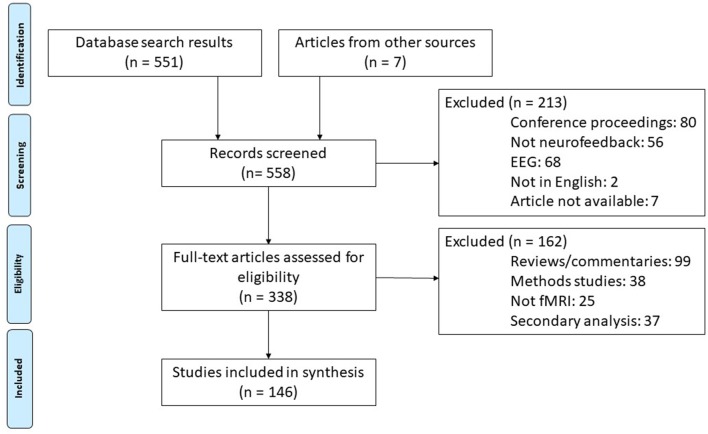
Diagram of literature search procedures across the primary and update literature search dates based on PRISMA 2009 Flow Diagram (Moher et al., 2009). fMRI, functional magnetic resonance imaging; rt-fMRI-NF, realtime functional magnetic resonance imaging neurofeedback training.

Our systematic review was conducted in accordance with PRISMA guidelines (Moher et al., 2009). We conducted a Web of Science search using the parameters “neurofeedback” AND “fMRI” on January 8th, 2019 and again on September 25th, 2019. We then screened out conference proceedings, those articles using an EEG modality, those not available in English, and others that were falsely identified as neurofeedback studies by the search engine. Of the remaining publications, we identified articles reporting original research studies. This excluded reviews, opinion pieces, methods only papers, and those using other non-fMRI modalities. During the process of reviewing the articles (described below), several additional research studies missed in the initial literature search were identified and added to the review. This resulted in 183 scientific publications identified as research reports. Articles reporting secondary analysis or otherwise reusing participant data were not included so as not to overrepresent single studies (final number of unique studies = 146). See Figure 2 for a flow chart of this literature search procedure.

Following identification of these reports, the study methods were evaluated on the following information: total sample size, type of sample (e.g., patient, healthy control), type of task used for neurofeedback (e.g., motor imagery, cue reactivity), type of neurofeedback calculation (e.g., signal from an ROI, SVM classification), type of regulation procedure (e.g., up/down regulation), type of feedback (e.g., thermometer, line graph), whether feedback was explicitly or implicitly related to changing brain activity, whether feedback was continuously or intermittently presented, type of control for active neurofeedback, how many runs of (i.e., how much) neurofeedback training per session, how many sessions of neurofeedback training with what interval, and how long of a follow-up was conducted to evaluate sustained affects (if applicable). Study results were evaluated on whether there was an effect of neurofeedback on ability to regulate brain activity and if neurofeedback translated to clinical or behavioral changes. This study synthesis process was conducted by two of the authors (S.F. and S.D.). A random 5% of the studies were double-rated by a third author (T.M.). Plots were generated using the “ggplot2” package (v2.2.1) for R (v3.4.2; Wickham, 2009).

## Current State of the rt-fMRI Neurofeedback Field

**Figure 3:**
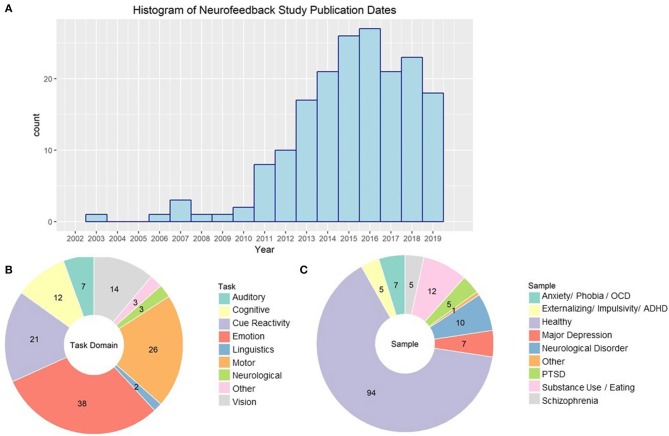
**(A)** Histogram of publication dates for all empirical rt-fMRI neurofeedback studies. **(B)** rt-fMRI neurofeedback studies categorized by task type. NA studies had no stimuli or instructions that fell into a particular task domain. Numbers indicate the number of studies in each category. **(C)** rt-fMRI neurofeedback studies by type of sample used in the study. Numbers indicate the number of studies in each category.

There has been a sharp increase in the number of publications concerning rt-fMRI neurofeedback, including both original research reports and reviews on the topic. In 2018, there were 23 papers published using rt-fMRI-NF methods. See Figure 3A for a histography of the number of studies published by year. This research generation will only grow. Based on a September 21st, 2019 query of current projects with “fMRI” and “neurofeedback” in the titles or abstract, NIH Report indicates that there are currently 25 research projects supported by the National Institutes of Health (primarily National Institute of Mental Health) and Veterans Affairs, with total funding of more than $12 million.

### Behavioral Domains Where Neurofeedback Has Been Investigated (Figure 3B)

Part of the appeal of neurofeedback may be its versatility as a tool to investigate a variety of neural and psychological processes. However, it has been most popular in emotion regulation applications. We found 38 unique published experiments using some form of emotion paradigm. Studies have demonstrated that individuals are able to up and down regulate their amygdala activity while viewing emotional pictures (Johnston et al., 2010; Hellrung et al., 2018; Herwig et al., 2019) and while listening to emotional music (Lorenzetti et al., 2018). Rt-fMRI neurofeedback training of the amygdala improves symptomology for disorders such as major depressive disorder (Young et al., 2017; MacDuffie et al., 2018), and post-traumatic stress disorder (Gerin et al., 2016; Zotev et al., 2018b). There is also initial evidence that it could be useful for individuals with borderline personality disorder (Paret et al., 2016).

Emotional reactivity, as well as craving, has also been investigated using cue-reactivity paradigms (21 published studies). These involve presenting pictures that are designed to induce craving or anxiety symptomology. For example, one study showed pictures of food and asked subjects to reduce activity in brain regions associated with craving (Ihssen et al., 2017). This has also been used to downregulate craving in individuals with AUD (Karch et al., 2015) and obesity (Spetter et al., 2017), as well as smokers (Hartwell et al., 2016) and cocaine users (Kirschner et al., 2018). Other studies present individuals with stimuli that invoke anxiety, such as trauma-inducing words for patients with PTSD (Nicholson et al., 2017), contamination images for patients with OCD (Scheinost et al., 2013b; Rance et al., 2018), and pictures of spiders in females with spider phobia (Zilverstand et al., 2015). By practicing control of reactivity based on signal from the orbitofrontal cortex (OFC), dorsolateral prefrontal cortex (dlPFC), and insula, these patients had reduction in anxiety symptomology.

Another field where extensive work has been done using rt-fMRI neurofeedback is motor function, with 26 published studies. These tasks typically involve either motor imagery or finger tapping paradigms (e.g., Berman et al., 2012) and involve primarily upregulation of primary motor or premotor/supplementary motor activity (SMA). However, there are mixed results in this field; for example, the previously cited study from Berman and colleagues found that primary motor cortex regulation was possible during finger tapping but not motor imagery, while Mehler et al. (2019) found that neurofeedback was associated with a decrease in primary motor but an increase in SMA engagement activity during motor imagery. Rt-fMRI-NF training focusing on upregulating the SMA during finger tapping and motor imagery is promising for reducing motor symptoms of Parkinson's (Subramanian et al., 2016) Huntington's (Papoutsi et al., 2018), and chronic stroke (Liew et al., 2016).

Several perceptual fields have also employed rt-fMRI neurofeedback. There are 14 published studies on vision and 7 on audition. Subjects were able to upregulate, and to a lesser extent downregulate (Cortese et al., 2017), visual region activity and connectivity with neurofeedback during visual perception and imagery tasks (e.g., Sousa et al., 2016). This transferred in some cases to changes in perceptual accuracy (Scharnowski et al., 2012). In fact, by using a neurofeedback technique designed to associate color-related brain states with visual perception of horizontal/vertical lines, Amano et al. (2016) were able to induce perception of color in its absence. Initial evidence suggests that control of visual perception aided by neurofeedback may be useful for controlling hallucinations in individuals with schizophrenia (Dyck et al., 2016) and improving attention in adolescents with ADHD (Alegria et al., 2017). Subjects have also been able to use neurofeedback to regulate activity in the primary and secondary auditory cortex both up (Yoo et al., 2007) and down (Emmert et al., 2017; Sherwood et al., 2018). Neurofeedback aided down-regulation of the auditory cortex has been associated with reduction in auditory hallucinations (Orlov et al., 2018) and tinnitus (Haller et al., 2010).

The ability to regulate activity associated with cognition with the aid of rt-fMRI neurofeedback have also been investigated, with 12 published studies using various tasks. Individuals are able to use neurofeedback to improve sustained attention (Debettencourt et al., 2015), working memory (Zhang et al., 2013), and visual memory (Hohenfeld et al., 2017). There may also be potential to use this technique in prodromal stages of Alzheimer's, individuals with schizophrenia (Cordes et al., 2015), adults with ADHD (Zilverstand et al., 2017), and major depressive disorder (Schnyer et al., 2015). Finally, individuals could both up and down regulate insula, and to a lesser extent, anterior cingulate (ACC) engagement during pain with the aid of rt-fMRI neurofeedback. However, there is contradictory evidence on whether control of this signal is associated with a reduction in perceived pain (Emmert et al., 2014) or not (Rance et al., 2014a).

### Investigation of rt-fMRI Neurofeedback Using Clinical Populations

Despite some work on applications to clinical populations, the majority of reviewed studies (64%) use healthy control samples (see Figure 3C). In many cases, this is to explore the feasibility of regulating a given neural response (e.g., Yoo and Jolesz, 2002) or to validate new methods (e.g., Koush et al., 2013). The most investigations into clinical populations are on anxiety, including PTSD, disorders where there is already demonstrated efficacy for EEG (Moore, 2000) and heart rate variability biofeedback (Goessl et al., 2017). Investigation of rt-fMRI neurofeedback training as an intervention for major depressive disorder has been published on primarily by two groups: Young et al. (2017) and Mehler et al. (2018).

The ability for substance users to regulate craving related-brain activity has been studied by a variety of groups. Use of patient samples in this field may be due to the face validity of instructions to lower symptomology and the more limited ability to create a non-patient experimental model. Heavy smokers and heavy drinkers, as well as cocaine users, were able to regulate brain activity and connectivity across a distributed salience and executive control network (Karch et al., 2015; Kim et al., 2015; Hartwell et al., 2016; Kirschner et al., 2018). However, this barely addresses the myriad of substance use disorder plaguing our communities, such as the opioid epidemic, and hasn't yet examined whether regulating craving translates to reduction in substance use.

Several other patient populations have had one or two studies dedicated toward them. These include rt-fMRI neurofeedback training designed decreased parietal connectivity in individuals with autism spectrum disorder (Ramot et al., 2017), increasing amygdala connectivity during emotional processing in borderline personality disorder (Paret et al., 2016), and increasing insula activation during emotion process in sex offenders, although only 25% of those individuals were successful (Sitaram et al., 2014). Neurologic disorders have also been investigated, including chronic stroke (Liew et al., 2016), Huntington's (Papoutsi et al., 2018), Parkinson's (Subramanian et al., 2016), neuralgia (Guan et al., 2015), and tinnitus (Haller et al., 2010).

There is much discussion of translations of the research using healthy samples to clinical applications, such as stroke (Wang et al., 2018), OCD (Goncalves et al., 2017), hallucinations (Fovet et al., 2016), motor rehabilitation (Linden and Turner, 2016), internet addiction (Becker and Montag, 2017), eating disorders (Sokunbi, 2018), and psychiatry in general (Arns et al., 2017). However, the studies that have been done comparing the efficacy of rt-fMRI neurofeedback in healthy vs. clinical samples suggests translation between the two samples is not that simple. For example, Cordes et al. (2015) found that schizophrenia patients and healthy controls had different cognitive strategies to upregulate the ACC, and that activity focused in different subregions of the ACC between the groups (dorsal and rostral, respectively). Severity of disease may also impact the ability to regulate with the aid of rt-fMRI neurofeedback. Patients with more severe PTSD were more able to downregulate their amygdala response to trauma words (Nicholson et al., 2017), while smokers with more severe nicotine dependence were less able to downregulate their ACC response to smoking cues (Canterberry et al., 2013). Moreover, older adults, who are more likely to need stroke or motor rehabilitation, may not respond as well to rt-fMRI neurofeedback as younger adults (Rana et al., 2016).

## Design Elements of rt-fMRI Neurofeedback Studies

Clearly, there is additional work necessary before rt-fMRI based neurofeedback can be considered a viable option for intervention with patients. A key element of this is to design trials that have the power and methodology to cause and detect clinically relevant effects. Understanding inconsistencies and establishing consensus for best practices going forward is essential. Moreover, by reviewing those areas that have been investigated thoroughly, we identify areas that are lacking empirical work. In the following sections, we summarize typical approaches to the essential elements of a neurofeedback training experiment and describing any evidence suggesting one approach is preferable to another.

### Algorithms and Sources of Neurofeedback Signal

Rt-fMRI neurofeedback works by providing a representation of hemodynamic response in the brain compared to some baseline, allowing subjects to implicitly learn to regulate their neural activity. However, a standard fMRI pulse sequence might collect signal from tens of thousands of sources. Defining which portion of that signal to feedback and display is one of the most important elements of designing a rt-fMRI neurofeedback study.

**Figure 4:**
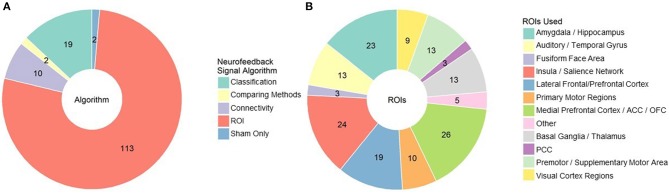
**(A)** rt-fMRI neurofeedback studies categorized by signal/algorithm type. Classification includes SVM, DecNef, MVPA etc. Numbers indicate the number of studies in each category. **(B)** rt-fMRI neurofeedback studies that employed an ROI signal approach by region used for that ROI. Numbers indicate the number of studies in each category. ROI, region of interest; ACC/PCC, anterior/posterior cingulate cortex; OFC, orbitofrontal cortex.

The first element of the source decision is what general algorithm/approach is most appropriate for your hypotheses and study design (Figure 4A). The most popular approach, used in 104 published studies to date, is to select an anatomical region of interest (ROI), average the signal across that region, and display it in some summary form. In some cases, experimenters can further individualize the anatomical ROI by identifying in a baseline scan for each subject where response corresponding to some function (e.g., craving) is located (e.g., Sorger et al., 2018). In a pilot study conducted by our group, we have also found that information from several ROIs can be weighted and combined to allow individuals to regulate craving related activity. The ROI approach is likely most appropriate when there is strong support for a specific and localized function of that anatomical region, and that function is directly related to the desired behavioral change. For example, a large portion of studies using the ROI approach involve regulation of the amygdala during emotion processing and cue reactivity (the literature on which we have summarized above).

There are many other anatomical locations that have been used for neurofeedback (see Figure 4B for a summary). Motor and visual regions are used, as you might expect, in motor imagery and visual perception tasks, respectively. Medial and lateral prefrontal regions, including the OFC and ACC, are some of the most common regions used as ROIs. Their role in self-regulation and integrating reward processes likely identify them as universally relevant to rt-fMRI neurofeedback, regardless of the target behavior. Salience network areas, particularly the insula, and basal ganglia regions such as the putamen and nucleus accumbens are also common targets, particularly for studies involving cue reactivity (e.g., Sokunbi et al., 2014). However, choosing a region when many are involved may lead to suboptimal reinforcement. For example, the inferior parietal lobule was a better ROI for neurofeedback than other visual association ROIs during visual imagery (Harmelech et al., 2015) and the primary motor cortex was not effective for motor imagery (Berman et al., 2012). It may be that we are not as good at selecting specific ROIs from the literature as we hope; neurofeedback based on automated selection of ROIs is just as effective as expert selected ROIs (Luhrs et al., 2017).

Related to the ROI approach, eight studies have taken the connectivity approach of feeding back functional connectivity signals, encouraging synchronizing or desynchronizing brain activity across regions. For example, Yamashita et al. (2017) demonstrated that subjects could increase or decrease connectivity between ipsilateral motor and parietal regions with the aid of rt-fMRI neurofeedback while Koush et al. (2015) found that subjects could upregulate connectivity between medial prefrontal cortex (mPFC) and amygdala regions of interest, corresponding to higher ratings of emotional arousal. This approach has been taken to normalize intraparietal connectivity in individuals with autism spectrum disorder (Ramot et al., 2017), and to increase cognitive control of eating behaviors in obese males via the dlPFC-mPFC pathway (Spetter et al., 2017).

There are also several approaches that aim to classify brain activity, and then provide feedback based on proximity to the desired brain state. These procedures have the advantage of capturing and incorporating more data from the whole brain and accounting for individual strategies and compensatory processes outside of the expected networks. Use of this approach is more recent given the additional processing power for realtime classification, but it should no longer be held back by this concern. The most common classifier used by experimenters for rt-fMRI neurofeedback are support vector machines (SVM) and other multivoxel pattern analysis (MVPA) approaches (e.g., Debettencourt et al., 2015). SVM is a supervised learning algorithm that calculates a hyperplane by which it can best distinguish between two sets of data spaces. When new cases (i.e., whole brain images) are given to it, it places each in one of the two classes based on that hyperplane, and computes the distance the new data case is from the hyperplane. As a result, SVM based rt-fMRI neurofeedback can provide a continuous scale representing the baseline vs. desired class on each end. For example, McDonald et al. (2017) classified mind wandering and focusing states in rt-fMRI neurofeedback and used that information to aid subjects in regulating their default mode network engagement. Another approach, called Decoded Neurofeedback (or DecNef), uses classification of brain activity to implicitly incept desired brain states (Shibata et al., 2011). In addition to the visual perceptual learning it was developed for, DecNef has been used to associate rewards with fear brain states, reducing amygdala and skin response to phobia cues in individuals with anxiety (Taschereau-Dumouchel et al., 2018).

Although limited work has been done examining the relative effectiveness of these feedback sources, there is some evidence to suggest that signal reflecting the interconnected nature of the brain is more effective than the single ROI approach. In a direct comparison, connectivity feedback was more effective than ROI feedback for regulation of cigarette craving (Kim et al., 2015). The preferability of using connectivity or whole-brain dynamics is intuitive given it mirrors the movement of the general field of fMRI from focusing on “blobs” of activity to dynamics of activity across the brain. Moreover, we know that multiple brain regions are involved in learning with rt-fMRI neurofeedback to regulate activity in a single brain region (Kopel et al., 2017). One study has conducted both classifier and ROI feedback, and found them comparable; notably however, that study did not report statistical comparison of the two groups (Lorenzetti et al., 2018). Given the lack of direct comparisons, we cannot provide a concrete recommendation. However, we do encourage individuals developing neurofeedback protocols to consider using a source/algorithm incorporating information from multiple regions in the brain, despite the preponderance of single ROI based approaches. We feel this approach is more consistent with the growing understanding of the importance of functional connectivity and multiple brain regions forming networks in complex behavior, as well as the reviewed evidence of support regions engaged during neurofeedback training. That being said, an ROI-based approach may be more appropriate for mechanistic studies, or when there is compelling evidence for strong and specific localization of target behavior.

#### Recommendation

Further research directly comparing sources/algorithms for neurofeedback signal in terms of clinical efficacy is needed.

**Figure 5:**
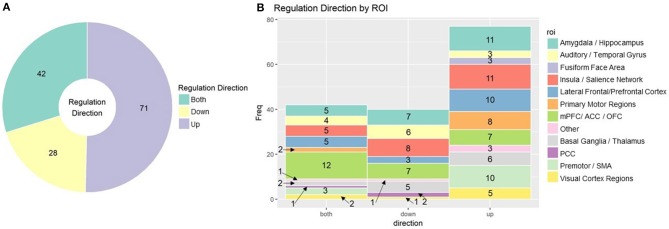
**(A)** rt-fMRI neurofeedback studies categorized by direction subjects were instructed to regulate Numbers indicate the number of studies in each category. **(B)** Stacked bar char of rt-fMRI neurofeedback studies using the ROI approach broken down by direction of regulation and ROI. Numbers indicate the number of studies in each category. ROI, region of interest; mPFC, medial prefrontal cortex; ACC/PCC, anterior/posterior cingulate cortex; OFC, orbitofrontal cortex; SMA, supplementary motor area.

More than just the location of the signal, the desired change in signal is an important element of designing rt-fMRI neurofeedback training protocols. Many studies ask subjects to regulate brain activity up and/or down, but upregulation has been given more focus in the field. Subjects may also be able to regulate to more than two levels of “up” or “down” (Sousa et al., 2016; Krause et al., 2017). This decision is likely to be ROI or function specific. In addition to theory driven considerations, subjects find it easier to upregulate activity in regions such as the visual cortex (Cortese et al., 2017) and the insula (Kadosh et al., 2016), but easier to downregulate the amygdala (Paret et al., 2018). In Figure 5, we summarize the proportion of up, down, and bidirectional regulation (Figure 5A) in rt-fMRI neurofeedback training studies with break down by ROI (Figure 5B).

#### Recommendation

The direction of regulation trained by neurofeedback should be context and region dependent.

### Feedback Displays and Instructions for Regulation Based on Feedback

**Figure 6:**
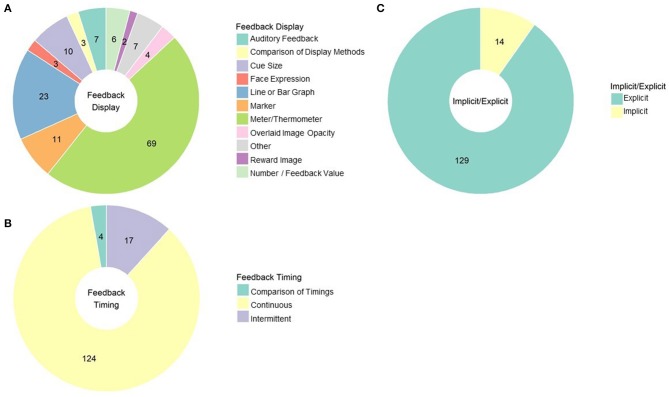
**(A)** rt-fMRI neurofeedback studies categorized by the way feedback was displayed to subjects. Numbers indicate the number of studies in each category. **(B)** rt-fMRI neurofeedback studies by feedback timing. Numbers indicate the number of studies in each category. **(C)** rt-fMRI neurofeedback studies by nature of the instructions for regulating brain activity. Numbers indicate the number of studies in each category.

The most common methods that experiments use to convey and present rt-fMRI based neurofeedback to their subjects are activity meters (69 studies) and continuous line or graphs (23 studies; see Figure 6A for a summary of these feedback displays). Many resemble vertical thermometer bars reflecting to subjects current level of activity only (sometimes compared to a baseline or goal line; e.g., MacInnes et al., 2016). Others provide a running account of neural response by plotting activity at each time point and connecting it to the previous time point via a line (e.g., Van De Ville et al., 2012). Others provide position markers that are not necessarily fixed to vertical or horizontal orientations, with feedback instructions to move the marker toward some target (e.g., Perronnet et al., 2017).

Other rt-fMRI neurofeedback studies employ more creative forms of feedback displays. One such approach is to display a cue that gets larger or smaller depending on neural engagement. This is a common approach to feedback display used in DecNef studies (Amano et al., 2016). It is also sometimes used in cue-reactivity studies to mirror approach-avoidance therapies (i.e., as cue reactivity is downregulated, the cue gets smaller; Ihssen et al., 2017). Another approach appropriate to classification methods is to change the color of the environment based emotion state (Lorenzetti et al., 2018) or to change the relative opacity of two overlapping pictures (faces and scenes) to reflect attentional focus (Debettencourt et al., 2015). Monetary reward for successful regulation may be a helpful reinforcer as well as the neurofeedback itself (Bray et al., 2007; Sepulveda et al., 2016).

There is some work that compares approaches to displaying neurofeedback. Social feedback (i.e., an avatar engaging with the subject) is more effective than standard feedback (Mathiak et al., 2015), for example, and displaying more than one feedback signal at once (i.e., two thermometers) leads to poorer regulation of neural response (Hartwell et al., 2013). Auditory feedback, such as changes in tones or in verbal reinforcement from the experimenter, may be more effective than visual feedback (Harmelech et al., 2013), although only 5 published studies have reported using auditory feedback. In fact, Harmelech and colleagues found that even visual and auditory feedback in combination was found to be less effective than auditory feedback. Subjects in that study reported that auditory feedback was less distracting than visual feedback from mental strategies. It could be imagined that in cue reactivity studies, auditory stimuli would also allow subjects to focus visually on the cue picture rather than the feedback source.

Regardless of the method of feedback rt-fMRI neurofeedback usually consists of delivering that feedback consistently through regulation trials; 111 published studies use the continuous feedback approach. However, some studies give feedback intermittently, such as a numeric summary score at the end of regulation blocks (Sarkheil et al., 2015) or a marker that moves only after a block of regulation (Baecke et al., 2015). Three of the four studies done comparing these approaches find that intermittent feedback is more effective than continuous feedback for enabling premotor cortex (Johnson et al., 2012) and amygdala regulation (Marxen et al., 2016; Hellrung et al., 2018). This is consistent with operant condition principles; variable and interval reinforcement schedules are more effective reinforcers than constant reinforcement. However, it should be noted that Emmert et al. (2017) reported continuous feedback was more effective than intermittent during auditory cortex regulation. See Figure 6B for the proportion of studies using these approaches.

#### Recommendation

Display feedback intermittently to best reinforce desired neural patterns, unless you are examining the auditory cortex; consider using auditory feedback, as opposed to visual feedback, when possible and appropriate.

Finally, the last major consideration when developing a feedback procedure is interpreting the feedback for the participant, either via context on the stimulus display or in instructions prior to starting the neurofeedback training. The vast majority of studies use explicit instructions, indicating that the feedback display represents changes in function specific brain activity (see Figure 6C for the proportion of studies using implicit and explicit instruction approaches). However, there are strong proponents of using implicit designs (e.g., asking subjects to complete a picture image without providing any guidance on how to do that), such as Ramot et al. (2016), who have shown that brain networks can be modified even without intention to change or awareness of it. Work from the same group hints that this implicit learning could have clinical applications; although they found no overall improvements in symptomology, changes in resting-state brain activity following neurofeedback were correlated with improvement in social responsiveness (Ramot et al., 2017). Implicit neurofeedback may be useful for reduction in fear response to conditioned aversive stimuli, which may have implications for PTSD or phobia treatment (Koizumi et al., 2017; Taschereau-Dumouchel et al., 2018), and classic DecNef studies also use implicit neurofeedback to aid perceptual learning without awareness of what was to be learned (Shibata et al., 2011; Amano et al., 2016).

On the other hand, Garrison et al. (2013) investigated effortful awareness with both implicit and explicit neurofeedback and found no difference in effectiveness for the two techniques. Other studies report that neurofeedback aids in acquisition of explicit strategies to regulate brain activity that can be clinically useful in the absence of feedback (Kopel et al., 2017), such as mindfulness techniques (Sherwood et al., 2018), positive words (Greer et al., 2014), and CBT strategies (MacDuffie et al., 2018). Although these cognitive effects may muddy the picture of whether neurofeedback itself improves outcomes, the clinical cost of using neurofeedback in the absence of clear evidence of its effectiveness supports the use of explicit instructions. This is especially relevant given some argument that the majority of the benefit of neurofeedback comes from placebo effect (Thibault and Raz, 2017). Denying patients the opportunity to benefit from this without evidence that implicit feedback is more effective may even be an ethical violation.

#### Recommendation

Within clinical applications, consider using explicit neurofeedback to help patients connect cognitive strategies and neuromodulation; in specific applications, such as reducing fear response, implicit neurofeedback may be more appropriate. Further research comparing techniques is necessary to evaluate the training performance differences of implicit vs. explicit neurofeedback.

### Experimental Design Considerations

**Figure 7:**
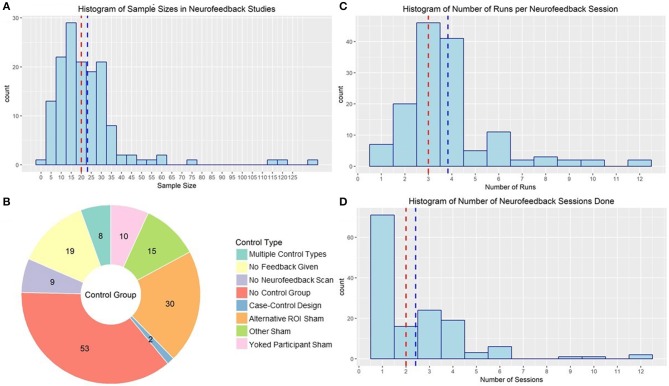
**(A)** Histogram of sample sizes used in rt-fMRI neurofeedback studies. Red line indicates median, blue line indicates mean. **(B)** rt-fMRI neurofeedback studies by nature of the control group used to compared the active neurofeedback condition. Numbers indicate the number of studies in each category. ROI, Region of interest. **(C)** Histogram of number of runs used in rt-fMRI neurofeedback training sessions. Red line indicates median, blue line indicates mean. **(D)** Histogram of number of sessions used in rt-fMRI neurofeedback training protocols. Red line indicates median, blue line indicates mean.

One of the largest problems with the rt-fMRI neurofeedback literature is the number of studies underpowered to detect the sort of transfer or group effects necessary to translate the technique to clinical practice. The median sample size in the reviewed studies was 20 subjects (mean: 23.07, sd: 18.95). Although sensitivity analysis studies of that size are powered to detect medium to large effect sizes (f = 0.33 | a = 0.05, Power = 0.8) in a simple 2 (pre/post) × 2 (neurofeedback/control) design, estimates of samples needed to detect clinical effects are as large as 101 subjects per group (Subramanian et al., 2016). As illustrated in Figure 7A, very few published studies have a total sample size >50. However, there is a positive correlation between year of publication and sample size, suggesting more recent studies are better powered to detect effects (r = 0.24, *p* = 0.004).

#### Recommendation

Plan recruitment of subjects for your rt-fMRI neurofeedback study such that you will be powered to detect sustained transfer effects.

Another concern when developing a rt-fMRI study is that neurofeedback has a clear placebo effect. There has been much discussion of this in the field of EEG-based neurofeedback, following findings that EEG sham neurofeedback was just as effective in treating insomnia as real neurofeedback (Schabus et al., 2017), underlining the importance of conducting rigorous double-blind placebo-controlled neurofeedback studies. There is also some evidence for placebo effect in rt-fMRI neurofeedback; studies have shown that subjects who think they are getting real feedback show changes in neural activation in many of the regions used as neurofeedback ROIs (Ninaus et al., 2013) and changes in reports of mood (Peciña et al., 2018). However, many published rt-fMRI neurofeedback studies lack an adequate control. Moreover, studies with control groups do report some changes in neural regulation in individuals not receiving rt-fMRI neurofeedback (Yoo et al., 2006; Kirsch et al., 2016), although not in all of the same regions or to the same extent as the active group. Despite this evidence of the necessity of employing a control group to be able to attribute effects to neurofeedback, 53 published studies did not have a proper control. These studies largely compared regulation within-subject to non-regulation blocks. Although there may be some value in neurofeedback training even if the clinical results are largely due to placebo effects (Thibault and Raz, 2017; Thibault et al., 2017), this lack of rigorous, randomized, double-blind control trials will continue to be a major detriment to the field if not addressed (Thibault et al., 2017).

There are several options for control groups, and little consensus on which is optimal (see Figure 7B for a summary of control strategies used by rt-fMRI neurofeedback studies). Many ROI studies use a second ROI of non-interest (i.e., that they don't expect to change given the function of the neurofeedback reinforcement). For example, Yao et al. (2016) used a signal from the average of a whole-brain slice distal from the target insula ROI as sham feedback for the control neurofeedback condition. Other studies used non-brain activity as the sham feedback signal (Zotev et al., 2018a) or used signal from another subject's active neurofeedback session (yoked neurofeedback; e.g., Hamilton et al., 2011). There were also no feedback control conditions, where subjects completed the scans and were presented the same stimuli but were not given any feedback signal (e.g., Johnston et al., 2011) or where subjects did not go through any version of the neurofeedback scan (e.g., Linden et al., 2012).

Early work suggests there may be no difference between control methodologies, although to our knowledge no specific work designed to evaluate their relative performance has been conducted (Caria et al., 2007, 2010). Thibault et al. (2018) advocate for placebo-control designs (i.e., including a sham neurofeedback group). Although we agree that properly controlled designs are a necessity and investigating the effect of neurofeedback above and beyond placebo effect is important, there is argument against using sham neurofeedback as well (Pigott et al., 2018). Neurofeedback training is built on operant conditioning principles in that we design feedback to reinforce desired neural response. Sham feedback entails withdrawing the reinforcer or applying a punisher even when the desired neural response is present, a process known to lead to extinction of a behavior. In other words, subjects may engage in the correct cognitive strategies and/or neural engagement, but see no change in their feedback signal, and conclude that strategy is ineffective. In addition to leading to potential over-estimate of the neurofeedback effect (e.g., by decreasing performance in the comparison group while increasing performance in the active group), there are ethical concerns for use of this approach in patient populations, where deconditioning may undermine treatment as usual. We should also note that of the two common sham feedback procedures, yoked feedback may be partially superior to ROI feedback in terms of not punishing desired behavior, if only because yoked feedback would reflect a realistic time course of learning the neural regulation skill.

As both sham and no neurofeedback controls have drawbacks, we should focus on using their respective advantages complementarily to maximize benefit. Sham neurofeedback controls for expectancy effects; no neurofeedback rules out an inflated effect size while still controlling for many of the psychological/cognitive effects of the treatment. This approach is further advantageous as it provides the field data to directly compare these control methods to improve methodology in the future.

#### Recommendation

Recruit two control groups for your neurofeedback experiment: a sham control and a no neurofeedback control.

The clinical impact of neurofeedback is also impacted by the number of runs and sessions of neurofeedback, but the ideal number is not yet established. The median neurofeedback protocol employed three neurofeedback runs in each of two neurofeedback sessions. However, 62 published neurofeedback studies only conducted one session (see Figures 7C,D for the distribution of number of runs and sessions). Many of these studies were interested in the general capacity of individuals to regulate (e.g., Yoo and Jolesz, 2002), making one session reasonable. The ability for one rt-fMRI neurofeedback session to make lasting changes to neural processes and symptomology is less credible. Limited work suggests that patients with schizophrenia benefited from two sessions of neurofeedback, and that a third did not add to their ability to regulate (Dyck et al., 2016), but Hohenfeld et al. (2017) found that older adults didn't improve in their ability to regulate after the first session. Neither of these studies were designed to evaluate the influence of number of sessions on sustained behavior or symptom changes at follow-up. More work needs to be done before we can begin to identify an evidence-based standard for number of rt-fMRI neurofeedback sessions.

#### Recommendation

Further research examining number of runs needed for clinical efficacy is needed.

### Outcome Measurements for rt-fMRI Neurofeedback

**Figure 8:**
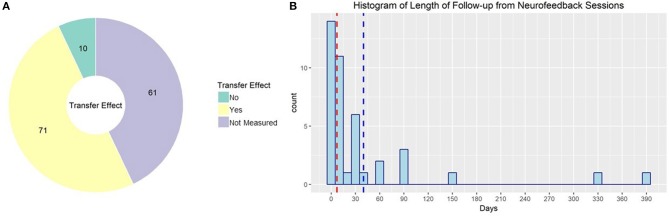
**(A)** rt-fMRI neurofeedback studies by nature of the outcome measure used to evaluate neurofeedback effectiveness. Numbers indicate the number of studies in each category. **(B)** Histogram of follow-up times used in rt-fMRI neurofeedback studies with follow-up sessions. Red line indicates median, blue line indicates mean.

Approximately half of studies published using rt-fMRI neurofeedback reported effects on behavior or symptomology; the rest only reported imaging results, although in some cases that included transfer effects to resting state or task fMRI (see Figure 8A for the proportion of studies evaluating non-imaging transfer effects). The primary outcome for the other half of these studies, however, was ability to regulate during neurofeedback and corresponding changes in brain activity. Although this is valuable information to form the base of our field, it is essential to demonstrate that the effect of rt-fMRI neurofeedback generalizes in a clinically useful way. Zilverstand et al. (2015) provide a good example. They found that in women with spider phobia, trained ability to downregulate their insula response during rt-fMRI neurofeedback predicted reduced spider fear at a 3 months follow-up.

Further complicating this issue, the length of time to follow-up may influence the magnitude of observed effects. In fact, several studies demonstrate that rt-fMRI neurofeedback effects are more pronounced at follow-ups of at least 2 months (Megumi et al., 2015; Rance et al., 2018). This continued improvement after neurofeedback training has been demonstrated in as little time as 1 day (Harmelech et al., 2013). Ability to regulate neural activity in a transfer task after rt-fMRI neurofeedback has been demonstrated to be maintained as long as 14 months (Robineau et al., 2017). Despite this, only 41 of the published studies collected and reported follow-up data (see Figure 8B for the distribution of time to follow up for these studies). Not only does this undercut the clinical relevance of rt-fMRI neurofeedback, it may lead to underestimates of the effect size of neurofeedback.

#### Recommendation

Collect assessments of clinical change post rt-fMRI neurofeedback training and during at least one follow-up visit.

## Other Concerns to Anticipate During Study Design

### Individual Differences

Many neurofeedback studies report that some portion of subjects receiving rt-fMRI neurofeedback are unable to regulate their neural response. Guan et al. (2015) and Banca et al. (2015) found that 75% of subjects could volitionally control their neural response with rt-fMRI neurofeedback training, while Robineau et al. (2014) and Chiew et al. (2012) found that only half of subjects could do so. However, one small study found that 1/4 subjects could successfully upregulate the insula (Sitaram et al., 2014), contributing to a sense of wide variation in ability to regulate across subjects (Hampson et al., 2012).

This variation may be in part driven by measurable individual differences. Individuals lower in agreeableness and less susceptible to anger are better able to regulate amygdala activity (Marxen et al., 2016). Higher levels of psychopathic traits relates to less ability to regulate the insula (Sitaram et al., 2014) while lower drive traits and performance on a monetary incentive delay task is associated with difficulty learning to regulate the nucleus accumbens during rt-fMRI neurofeedback (Li et al., 2018). However, neurofeedback-based regulation ability is likely not a universal trait and varies across regions of the brain (Rance et al., 2014a,b). As such, it is important to continue to investigate those traits that indicate what patients might be most responsive to a rt-fMRI neurofeedback-based intervention. In general, however, attention, motivation and mood are important influencers of neurofeedback performance (Kadosh and Staunton, 2019).

#### Recommendation

Collect personality and other trait measurements at baseline in your rt-fMRI neurofeedback study; consider selectively including those high in drive and lower in susceptibility to anger; account for a 25% rate of non-responders in your recruitment goals.

### Artifacts and Technical Problems

Rt-fMRI neurofeedback is becoming more accessible but may still require a fair amount of technical expertise to set up on a new site. There are several software packages used to process and send neurofeedback signals. We (among other groups) use the RT plugin and the 3dSVM plugin (LaConte, 2011) for AFNI (Cox, 1996) implemented in Python (available at github.com/afni/afni/tree/master/src/roopchansinghv/PsychoPy-NeuroFeedback-Demo). There are other widely used options, including Turbo-Brain Voyager (brainvoyager.com/products/turbobrainvoyager.html; Weiskopf et al., 2004), a BioImageSuite extension (bisweb.yale.edu; Scheinost et al., 2013a), a FSL based toolbox, FRIEND (nitrc.org/projects/friend/; Sato et al., 2013; Basilio et al., 2015), and an SPM/Matlab based toolbox OpenNFT (opennft.org; Koush et al., 2017). Many groups use custom implementations or extensions of these toolboxes and plugins. However, the choice between these options may come down to your habitual software choice for offline fMRI analysis.

Given the realtime nature of neurofeedback, processing typically doesn't involve the same extent of realtime processing as offline fMRI analysis. As such, there are some concern about the influence of signal of non-interest. Artifacts associated with eye movement (Zhang et al., 2011), respiration (Marxen et al., 2016), and sleeping (McDonald et al., 2017) did reduce the size of rt-fMRI neurofeedback training effects. On the other hand, motion artifacts did not drive classification-based neurofeedback results, although they may have influenced them slightly (Magland and Childress, 2014).

Neurofeedback has flexibility in terms of the characteristics of the fMRI scanner. Most published studies were conducting using a 3 Tesla machines, but it has also been successful on 1.5 Tesla (Gerin et al., 2016) and 7 Tesla scanners (Van den Boom et al., 2018). However, signal from these scanners may not be appropriate to compare between subjects given differences in amplitudes (Baecke et al., 2015), although it can be done simultaneously at different sites and magnet strengths with success. Siemens, Phillips, and GE scanners have all been used successfully for rt-fMRI Neurofeedback. It has not yet been investigated how differences in resolution and pulse timing, which would impact the specificity and frequency of the feedback given to the subject, influences outcomes associated with rt-fMRI neurofeedback training.

## Limitations to This Systematic Review

As with all reviews of the literature, there is a publication bias toward positive results in the studies published here. In fact, we found no published studies that reported no individuals successfully able to regulate neural activity with the aid of rt-fMRI neurofeedback training. However, 18% of studies reported mixed results such as null transfer effects or null effects for some ROIs but not others. Three studies that reported assessment of symptomology or behavior post neurofeedback training had null results. Moreover, it is possible we missed rt-fMRI neuroimaging studies that were not indexed or otherwise weren't found given our search parameters; however, we believe the sample of studies we have identified is generally representative of the field.

We did not review studies from other modalities in this review. EEG-based neurofeedback has been extensively reviewed and is accepted in clinical practice. We do not comment on how rt-fMRI neurofeedback compares to EEG neurofeedback in effectiveness. However, we will note that several studies have provided combined EEG/rt-fMRI feedback (Zotev et al., 2014), and have reported the multimodal approach is even more effective for reinforcing desired neural response (Perronnet et al., 2017). There have also been investigations into neurofeedback using magnetoencephalography (e.g., Okazaki et al., 2015), function near-infrared spectroscopy (e.g., Kober et al., 2015), and transcranial doppler ultrasound (Rey et al., 2018).

We used a single database, Web of Science, to systematically search the literature for publications on rt-fMRI neurofeedback to include in this review. Although we supplemented these with others encountered during the process of synthesizing the literature, PRISMA guidelines recommend searching multiple databases (Moher et al., 2009). It is possible this review failed to incorporate articles that would have been captured if multiple databases were used.

## Conclusions

The technique of rt-fMRI neurofeedback training is a safe and feasible approach to regulating brain activity and corresponding behavior. However, more rigorous, clinically focused studies are needed before it can be considered a viable intervention option. To contribute toward this goal, we have proposed recommendations based on the literature for each of the neurofeedback study design steps (summarized in Table 1). We believe these approaches will produce a rt-fMRI-NF study design that is mostly likely to elicit reproducible and consistent effects of neurofeedback. Although there remain unanswered questions, such as the effectiveness of rt-fMRI neurofeedback training in combination with other treatments, this is a necessary step toward transitioning from a focus on proof-of-concept studies to randomized controlled trials and ultimately clinical applications.

**Table 1:** Summary of recommendations for study design.

**Design element**	**Recommendation**
Algorithms and sources of neurofeedback signal	Further research directly comparing sources/algorithms for neurofeedback signal in terms of clinical efficacy is needed; The direction of regulation trained by neurofeedback should be context and region dependent.
Feedback display	Display feedback intermittently to best reinforce desired neural patterns, unless you are examining the auditory cortex; consider using auditory feedback, as opposed to visual feedback, when possible, and appropriate.
Instruction for regulation	Within clinical applications, consider using explicit neurofeedback to help patients connect cognitive strategies and neuromodulation; in specific applications, such as reducing fear response, implicit neurofeedback may be more appropriate. Further research comparing techniques is necessary to evaluate the training performance differences of implicit vs. explicit neurofeedback.
Sample size	Plan recruitment of subjects for your rt-fMRI neurofeedback study such that you will be powered to detect sustained transfer effects; use patient groups when possible.
Control	Recruit two control groups for your neurofeedback experiment: a sham control and a no neurofeedback control.
Number of sessions for training	Further research examining number of runs needed for clinical efficacy is needed.
Outcomes	Collect assessments of clinical change post rt-fMRI neurofeedback training and during at least one follow-up visit.
Software	AFNI, Turbo-Brain Voyager, and BioImage Suite are commonly used; more research is needed to determine differences between the implementations.
Other	Collect personality and other trait measurements at baseline in your rt-fMRI neurofeedback study; consider selectively including those high in drive and lower in susceptibility to anger; account for a 25% rate of non-responders in your recruitment goals.

## Data Availability Statement

The raw data supporting the conclusions of this article will be made available by the authors, without undue reservation, to any qualified researcher.

## Author Contributions

The concept for this article was created by SF and RM. Initial database searches and screening of the results was done by SF. SF and SD reviewed full-text articles for the information specified in the articles. TM reviewed a portion of the full-text articles as a double-rater. SF wrote the first draft of the manuscript, and RM provided substantive input on the manuscript. All authors contributed toward editing and approving the final manuscript.

### Conflict of Interest

The authors declare that the research was conducted in the absence of any commercial or financial relationships that could be construed as a potential conflict of interest.
